# Supplementary material for: Potentially Avoidable Hospitalizations by Asthma and COPD in Switzerland from 1998 to 2018: A Cross-Sectional Study
Source: Healthcare (Basel). 2023 Apr 26;11(9):1229. doi: 10.3390/healthcare11091229 (PMC10178069; doi:10.3390/healthcare11091229)
Supplement: Supplementary file 1 [file healthcare-11-01229-s001.zip › healthcare-2331875-supplementary.pdf]

Supplementary materials

Supplementary Table S1. Swiss cantons listed by the seven regions used for the study.

Region	Cantons
Leman	Geneva, Vaud, Valais
Mittelland	Bern, Solothurn, Fribourg, Neuchâtel, Jura
Northwest	Basel-Stadt, Basel-Landschaft, Aargau
Zurich	Zürich
Eastern	St. Gallen, Thurgau, Appenzell Innerrhoden, Appenzell Ausserrhoden, Glarus, Schaffhausen, Graubünden
Central	Uri, Schwyz, Obwalden, Nidwalden, Lucerne, Zug
Ticino	Ticino

Supplementary Table S2. Inclusion and exclusion criteria to define a potentially avoidable hospitalization for asthma.

**Inclusion criteria §**	
ICD-10-WHO	J450 PREDOMINANTLY ALLERGIC ASTHMA<br>J451 NONALLERGIC ASTHMA<br>J458 MIXED ASTHMA<br>J459 ASTHMA, UNSPECIFIED<br>J46 STATUS ASTHMATICUS
**Exclusion criteria †**	
ICD-10-WHO	E840 CYSTIC FIBROSIS WITH PULMONARY MANIFESTATIONS<br>E841 CYSTIC FIBROSIS WITH INTESTINAL MANIFESTATIONS<br>E848 CYSTIC FIBROSIS WITH OTHER MANIFESTATIONS<br>E849 CYSTIC FIBROSIS, UNSPECIFIED<br>P27.0 WILSON-MIKITY SYNDROME<br>P27.1 BRONCHOPULMONARY DYSPLASIA ORIGINATING IN THE PERINATAL PERIOD<br>P27.8 OTHER CHRONIC RESPIRATORY DISEASES ORIGINATING IN THE PERINATAL PERIOD<br>P27.9 UNSPECIFIED CHRONIC RESP DISEASE ORIGINATING IN THE PERINATAL PERIOD<br>Q25.4 OTHER CONGENITAL MALFORMATIONS OF AORTA<br>Q31.1 CONGENITAL SUBGLOTTIC STENOSIS<br>Q31.2 LARYNGEAL HYPOPLASIA<br>Q31.3 LARYNGOCELE<br>Q31.5 CONGENITAL LARYNGOMALACIA<br>Q31.8 OTHER CONGENITAL MALFORMATIONS OF LARYNX<br>Q31.9 CONGENITAL MALFORMATION OF LARYNX, UNSPECIFIED<br>Q32.0 CONGENITAL TRACHEOMALACIA<br>Q32.1 OTHER CONGENITAL MALFORMATIONS OF TRACHEA<br>Q32.2 CONGENITAL BRONCHOMALACIA<br>Q32.3 CONGENITAL STENOSIS OF BRONCHUS<br>Q32.4 OTHER CONGENITAL MALFORMATIONS OF BRONCHUS<br>Q33.0 CONGENITAL CYSTIC LUNG<br>Q33.1 ACCESSORY LOBE OF LUNG<br>Q33.2 SEQUESTRATION OF LUNG<br>Q33.3 AGENESIS OF LUNG<br>Q33.4 CONGENITAL BRONCHIECTASIS<br>Q33.5 ECTOPIC TISSUE IN LUNG<br>Q33.6 HYPOPLASIA AND DYSPLASIA OF LUNG<br>Q33.8 OTHER CONGENITAL MALFORMATIONS OF LUNG<br>Q33.9 CONGENITAL MALFORMATION OF LUNG, UNSPECIFIED<br>Q34.0 ANOMALY OF PLEURA

	Q34.1 CONGENITAL CYST OF MEDIASTINUM<br>Q34.8 OTHER SPECIFIED CONGENITAL MALFORMATIONS OF RESPIRATORY SYSTEM<br>Q34.9 CONGENITAL MALFORMATION OF RESPIRATORY SYSTEM, UNSPECIFIED<br>Q39.0 ATRESIA OF OESOPHAGUS WITHOUT FISTULA<br>Q39.1 ATRESIA OF OESOPHAGUS WITH TRACHEO-OESOPHAGEAL FISTULA<br>Q39.2 CONGENITAL TRACHEO-OESOPHAGEAL FISTULA WITHOUT ATRESIA<br>Q39.3 CONGENITAL STENOSIS AND STRICTURE OF OESOPHAGUS<br>Q39.4 OESOPHAGEAL WEB<br>Q39.8 OTHER CONGENITAL MALFORMATIONS OF OESOPHAGUS<br>Q89.3 SITUS INVERSUS
- Cases where the patient died in hospital during the admission<br>- Cases resulting from a transfer from another acute care institution (transfers-in)<br>- Pregnancy, childbirth, and puerperium<br>- Same day/day only admissions	

§, based on ICD-10 codes for the main cause of admission;

†, based on ICD-10 codes for secondary diseases or comorbidities. According to OECD Health Care Quality Indicators Project criteria [OECD. Health Care Quality and Outcomes (HCQO) 2018-19 Indicator definitions. [cited 2023 21.03.2023]; Available from: http://www.oecd.org/els/health-systems/Definitions-of-Health-Care-Quality-Outcomes.pdf].

Supplementary Table S3. Inclusion and exclusion criteria to define a potentially avoidable hospitalization for chronic obstructive pulmonary disease.

**Inclusion criteria §**	
ICD-10-WHO	J40 BRONCHITIS*<br>J410 SIMPLE CHRONIC BRONCHITIS<br>J411 MUCOPURULENT CHRONIC BRONCHITIS<br>J418 MIXED SIMPLE AND MUCOPURULENT CHRONIC BRONCHITIS<br>J42 UNSPECIFIED CHRONIC BRONCHITIS<br>J430 MACLEOD'S SYNDROME<br>J431 PANLOBULAR EMPHYSEMA<br>J432 CENTRILOBULAR EMPHYSEMA<br>J438 OTHER EMPHYSEMA<br>J439 EMPHYSEMA, UNSPECIFIED<br>J440 COPD WITH ACUTE LOWER RESPIRATORY INFECTION<br>J441 COPD WITH ACUTE EXACERBATION, UNSPECIFIED<br>J448 OTHER SPECIFIED CHRONIC OBSTRUCTIVE PULMONARY DISEASE<br>J449 CHRONIC OBSTRUCTIVE PULMONARY DISEASE, UNSPECIFIED<br>J47 BRONCHIECTASIS<br>* Qualifies only if accompanied by secondary diagnosis of J41, J43, J44, J47
**Exclusion criteria**	
- Cases where the patient died in hospital during the admission<br>- Cases resulting from a transfer from another acute care institution (transfers-in)<br>- Pregnancy, childbirth, and puerperium<br>- Same day/day only admissions	

§, based on ICD-10 codes for the main cause of admission;

According to OECD Health Care Quality Indicators Project criteria [13. OECD. Health Care Quality and Outcomes (HCQO) 2018-19 Indicator definitions. [cited 2021 08.01.2021]; Available from: http://www.oecd.org/els/health-systems/Definitions-of-Health-Care-Quality-Outcomes.pdf.].

Supplementary Table S4. Values of the diagnosis-related group point for 24 cantons of Switzerland.

Canton	Year	Value (CHF)
AG	2020	9,600.00
AI	2020	9,480.00
AR	2020	9,555.00
BE	2020	10,800.00
BL	2020	9,733.00
BS	2020	10,160.00
FR	2020	9,092.00
GE	2020	10,650.00
GL	2020	9,710.00
GR	2019	9,640.00
JU	2020	9,650.00
LU	2020	9,800.00
NE	2020	9,650.00
NW	2020	10,650.00
OW	2019	9,635.00
SG	2020	9,900.00
SH	2020	9,595.00
SO	2020	9,650.00
SZ	2020	9,772.00
UR	2020	10,650.00
VD	2020	10,650.00
VS	2020	9,550.00
ZG	2020	10,840.00
ZH	2020	10,855.00

1 CHF=1.01 EUR or 1.07 USD as of 16.03.2023
